# Long-Term Changes Following Orthopedic Mandibular Advancement Therapy in Pediatric Obstructive Sleep Apnea: a 7-Year Follow-up Study

**DOI:** 10.1007/s00784-026-06926-4

**Published:** 2026-05-25

**Authors:** Serife Kiran Aydil, Esen Kiyan, Refika Ersu, Hulya Kilicoglu, Gulsilay Sayar, Eser Capan

**Affiliations:** 1 Department of Orthodontics, Faculty of Dentistry, Istanbul University, Istanbul, Turkey; 2 Department of Pulmonary Medicine, Faculty of Medicine, Istanbul University, Istanbul, Turkey; 3 Children’s Hospital of Eastern Ontario Research Institute, University of Ottawa, Ottawa, Canada; 4 Department of Orthodontics, Faculty of Dentistry, Bahcesehir University, Istanbul, Turkey; 5 Private Practice Orthodontist, Nisantasi, Istanbul, Turkey

**Keywords:** Pediatric OSA, Mandibular retrognathia, Functional appliance, Long-term changes, Sleep

## Abstract

**Study Objectives:**

To evaluate long-term changes in craniofacial morphology, upper airway dimensions, and sleep-related parameters following monoblock mandibular advancement therapy in children with obstructive sleep apnea (OSA).

**Methods:**

This longitudinal follow-up study included 13 children (mean age 10.97 ± 1.51 years) with OSA and mandibular retrognathia treated with a monoblock appliance. The mean duration of active treatment was 8.06 ± 1.29 months. Assessments were conducted at baseline (T1), post-treatment (T2), and 7 years after treatment (T3), including lateral cephalometric radiographs, polysomnography (PSG), and Pittsburgh Sleep Quality Index (PSQI) scores. Pharyngeal airway changes were analyzed using a standardized cephalometric protocol across 11 sagittal reference planes. Repeated measures ANOVA and nonparametric equivalents were applied for longitudinal comparisons.

**Results:**

Increases in oropharyngeal and hypopharyngeal airway dimensions (S6–S10; referring to standardized sagittal measurement planes) observed after treatment were generally maintained over time. Skeletal parameters showed relative stability at follow-up. Although Apnea-Hypopnea Index (AHI) decreased significantly after treatment, an increase was noted at T3. The percentage of REM (Rapid Eye Movement) sleep showed no significant change between T1 and T2, whereas a statistically significant increase was observed at T3. PSQI scores improved initially and did not show significant long-term deterioration.

**Conclusions:**

This study provides long-term follow-up data on craniofacial, airway, and sleep-related changes in children with OSA treated with a monoblock appliance. Although partial recurrence of AHI was observed during adolescence, airway dimensions and skeletal parameters demonstrated relative stability over time. These findings suggest that structural adaptations may be maintained, while functional outcomes may vary during growth. Given the multifactorial nature of pediatric OSA, these results should be interpreted cautiously and warrant further investigation in larger longitudinal studies.

## Introduction

Sleep-disordered breathing (SDB) includes a spectrum of conditions from habitual snoring to obstructive sleep apnea (OSA), the most severe form, characterized by intermittent upper airway obstruction during sleep [1]. OSA is associated with multiple factors such as adenotonsillar hypertrophy, reduced muscle tone, and craniofacial anomalies (e.g., transverse maxillary deficiency, mandibular retrognathia, micrognathia, macroglossia) [2, 3]. These anatomical deviations may reduce airway dimensions and contribute to OSA [4, 5].

Pediatric OSA is increasingly recognized as a multifactorial disorder in which skeletal, neuromuscular, and behavioral components interact to influence airway patency [6]. Given its multifactorial nature, the optimal treatment for OSA remains debated. While adenotonsillectomy remains the first-line treatment for pediatric OSA and is effective in many cases, concerns about long-term outcomes and recurrence in certain patient populations have prompted interest in alternative or adjunctive therapies [7, 8].

Since abnormal mandibular and maxillary growth contributes significantly to pediatric OSA [4, 5], orthodontic treatments targeting these issues—such as rapid maxillary expansion [9], mandibular advancement appliances [10], and maxillary protraction devices [11]—have been explored.

Several studies have explored the long-term impact of these devices, particularly their ability to sustain mandibular advancement and cephalometric gains in airway dimensions in growing children [12–15]. Recent systematic reviews and meta-analyses have provided more comprehensive evidence on the effectiveness of mandibular advancement appliances in children with OSA, documenting significant reductions in AHI and improvements in oxygenation following treatment, although the overall evidence base remains limited by small sample sizes, heterogeneous designs, and short follow-up periods [16–18]. However, considering that OSA is influenced by not only skeletal parameters but also by neuromuscular, behavioral, and positional factors [19], evaluating only craniofacial structures may not provide a comprehensive understanding of the disorder in the long term. While some studies have examined the relationship between mandibular advancement and airway or polysomnographic outcomes [20–22], long-term follow-up studies combining both polysomnographic data and cephalometric airway measurements specifically in children remain scarce. Therefore, this study aims to evaluate the long term (7 years) changes of children with mandibular retrognathia and OSA who were treated with a monoblock appliance, by assessing both polysomnographic and cephalometric variables.

## Materials and Methods

This study builds upon a previous investigation [23] in which T1 (pre-treatment) and T2 (post-treatment) data were collected from patients diagnosed with OSA via polysomnography (PSG). The original study was conducted within a longitudinal framework. Accordingly, the present study represents a follow-up assessment of the same cohort, conducted 7 years after treatment completion (T3).

Ethical approval for the present study was obtained from the Human Ethics Committee of Istanbul Medipol University, Istanbul, Turkey (Approval No: 10840098-604.01.01.01-E.65336).

The initial cohort consisted of 16 consecutive pediatric patients with confirmed OSA (defined as an apnea–hypopnea index [AHI] ≥ 1) and skeletal Class II malocclusion (SNB angle < 78°, representing the sagittal position of the mandible relative to the cranial base) [23]. In the original study [23], exclusion criteria included cleft lip and/or palate, obesity, cardiopulmonary diseases, syndromic conditions, dysmorphism, and other craniofacial anomalies. Patients with clinically significant adenotonsillar hypertrophy were considered candidates for surgical management and were referred for adenotonsillectomy; such patients were not included. Consequently, none of the included patients had significant adenotonsillar hypertrophy or underwent adenotonsillectomy during the study period.

**Fig. 1:**
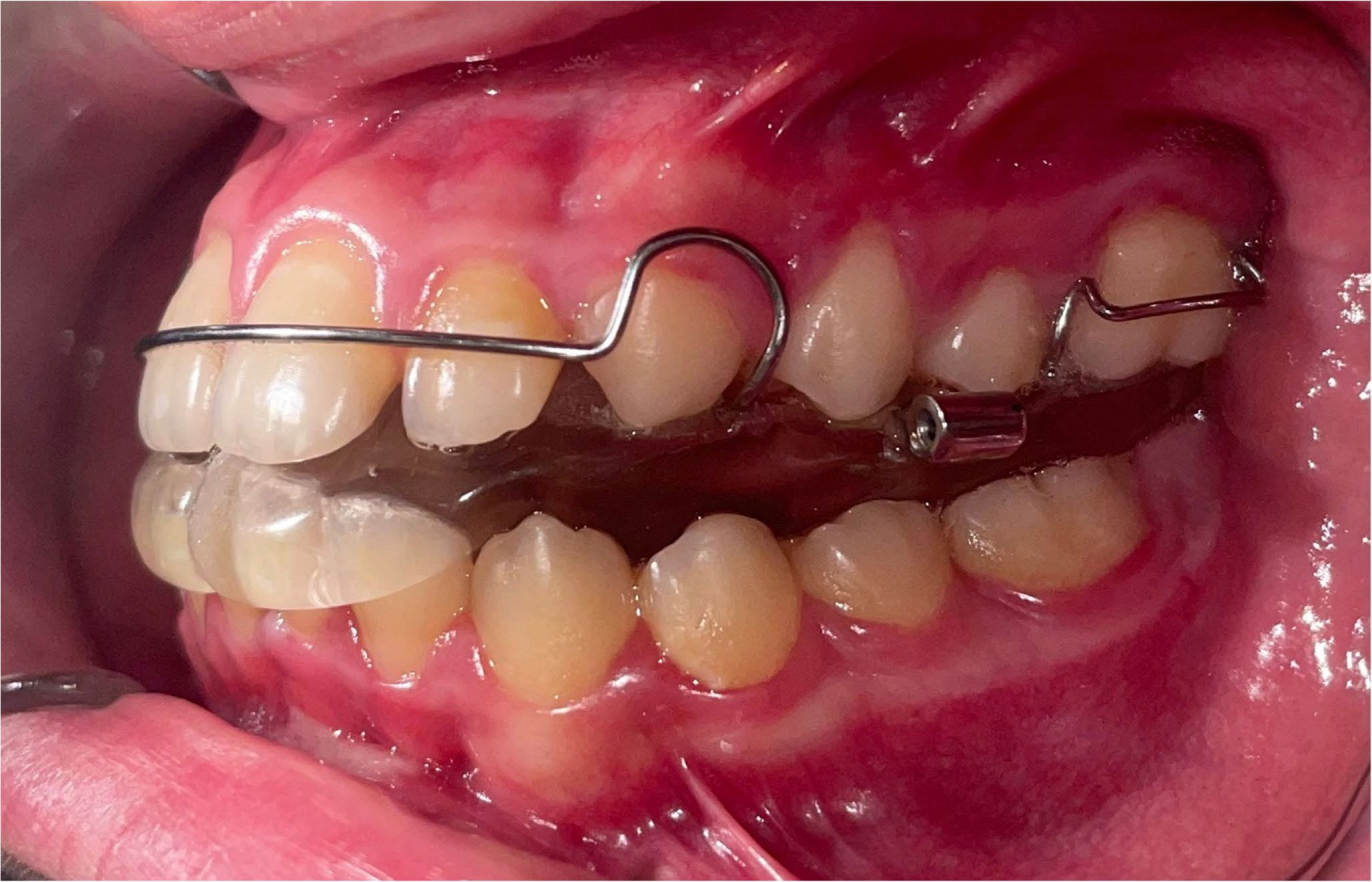
Design and intraoral view of monoblock appliance

Orthopedic mandibular advancement was selected due to mandibular retrognathia and confirmed OSA following interdisciplinary evaluation. Each patient was treated with an individualized monoblock appliance (Fig. 1), a functional orthopedic device commonly used to achieve mandibular advancement in growing patients [24]. The appliance covered the palatal and occlusal surfaces of the maxillary teeth and the occlusal and palatolingual surfaces of the mandibular teeth. Patients were instructed to wear the appliance for a minimum of 17 h per day. Treatment was discontinued once a stable Class I molar relationship was achieved, typically within 6–10 months.

All patients were invited to participate in the present follow-up study conducted 7 years after treatment completion. One patient declined participation, and two were excluded due to obesity based on reassessed body mass index (BMI) values. These excluded individuals did not differ from the remaining participants in terms of baseline age, craniofacial characteristics, or initial AHI values. Consequently, 13 patients (81% of the original cohort; 6 boys, 7 girls) were included in the final analysis. At treatment initiation, the mean age was 10.97 ± 1.51 years, and at treatment completion it was 11.64 ± 1.49 years. At the 7-year follow-up (T3), all participants had reached late adolescence or early adulthood.

Patients were considered suitable for monoblock therapy based on interdisciplinary clinical evaluation of airway patency and their ability to tolerate oral appliance use. All patients were assessed by otorhinolaryngology specialists at baseline and during follow-up visits. Adenotonsillar size and other upper airway conditions were evaluated using standard clinical grading methods. However, these variables were not systematically recorded in a standardized format or incorporated into the study database for quantitative analysis.

**Fig. 2:**
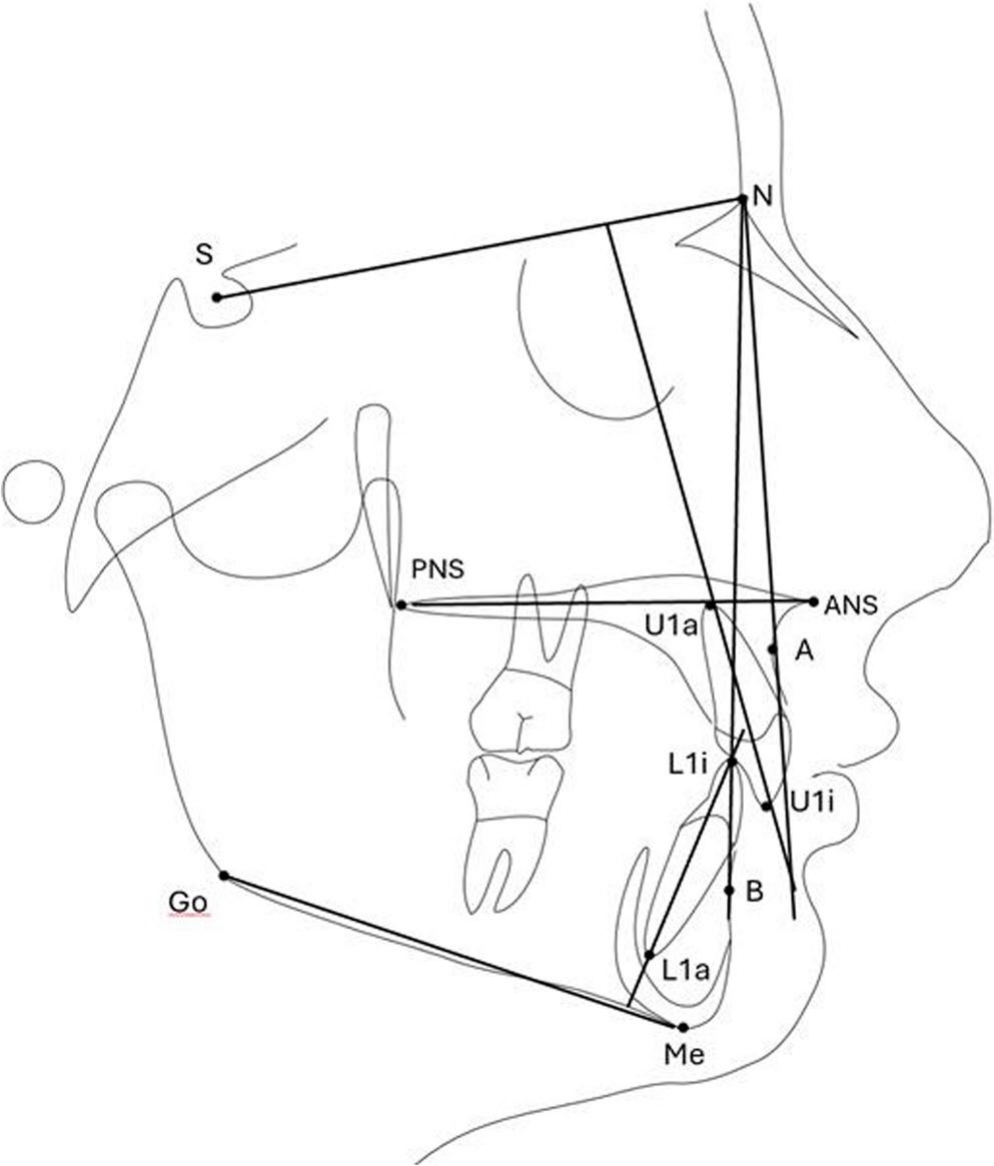
Cephalometric landmarks and reference lines used for skeletal and dental measurements. N, Nasion; S, Sella; ANS, anterior nasal spine; PNS, posterior nasal spine; Go, Gonion; Me, Menton (chin); A, the deepest concavity of the anterior maxilla; B, the deepest concavity of the anterior mandible; U1a, apex of the upper central incisor root; U1i, incisal tip of the upper central incisor; L1a, apex of the lower central incisor root; L1i, incisal tip of the lower central incisor

Lateral cephalometric radiographs, PSG, and Pittsburgh Sleep Quality Index (PSQI) assessments were conducted at three time points: pre-treatment (T1), post-treatment (T2), and at the 7-year follow-up (T3). The radiographic images were obtained under standardized conditions (62 kVp, 8 mA, 14.1 s) using the same device (Sirona Orthophos XG Plus DS/Ceph, Bensheim, Germany), with the patient in natural head position, teeth in maximum intercuspation, and lips relaxed. The radiographs were analyzed using NemoCeph Software (Nemotec, Madrid, Spain). All cephalometric tracings were conducted by the same orthodontist (E.C) to ensure consistency. The analysis was based on pharyngeal anteroposterior width, as well as sagittal and vertical skeletal parameters. Six linear and five angular cephalometric measurements were used in the study. These measurements, along with their definitions, are presented in Table 1 and illustrated in Fig. 2.

**Table 1:** Summary of Cephalometric Landmarks and Definitions

	Abbreviation	Definition
1	SNA (°)	Angle between the lines from Sella to Nasion and from Nasion to Point A; represents the maxillary position relative to the cranial base.
2	SNB (°)	Angle between the lines from Sella to Nasion and from Nasion to Point B; represents the mandibular position relative to the cranial base.
3	Go-Me (mm)	Linear distance between Gonion and Menton (chin); represents the length of the mandibular body.
4	ANS-PNS (mm)	Linear distance from Anterior Nasal Spine to Posterior Nasal Spine; indicates maxillary length.
5	SN-GoMe (°)	Angle between Sella–Nasion and Gonion–Menton lines; reflects the vertical growth pattern of the mandible.
6	1-SN (°)	Angle between the long axis of the upper central incisor (U1i to U1a) and the Sella–Nasion line; indicates upper incisor inclination.
7	1-NA (mm)	Linear distance from the most labial point of the upper incisor to the Nasion–Point A line; indicates upper incisor protrusion.
8	IMPA (°)	Angle between the long axis of the lower incisor (L1i to L1a) and the mandibular plane (Go–Me); measures lower incisor inclination.
9	1-NB (mm)	Linear distance from the most labial point of the lower incisor to the Nasion–Point B line; indicates lower incisor protrusion.
10	Overjet (mm)	Horizontal distance between the upper and lower incisal edges; indicates sagittal incisor relationship.
11	Overbite (mm)	Vertical overlap between the upper and lower incisal edges; indicates vertical incisor relationship.

**Fig. 3:**
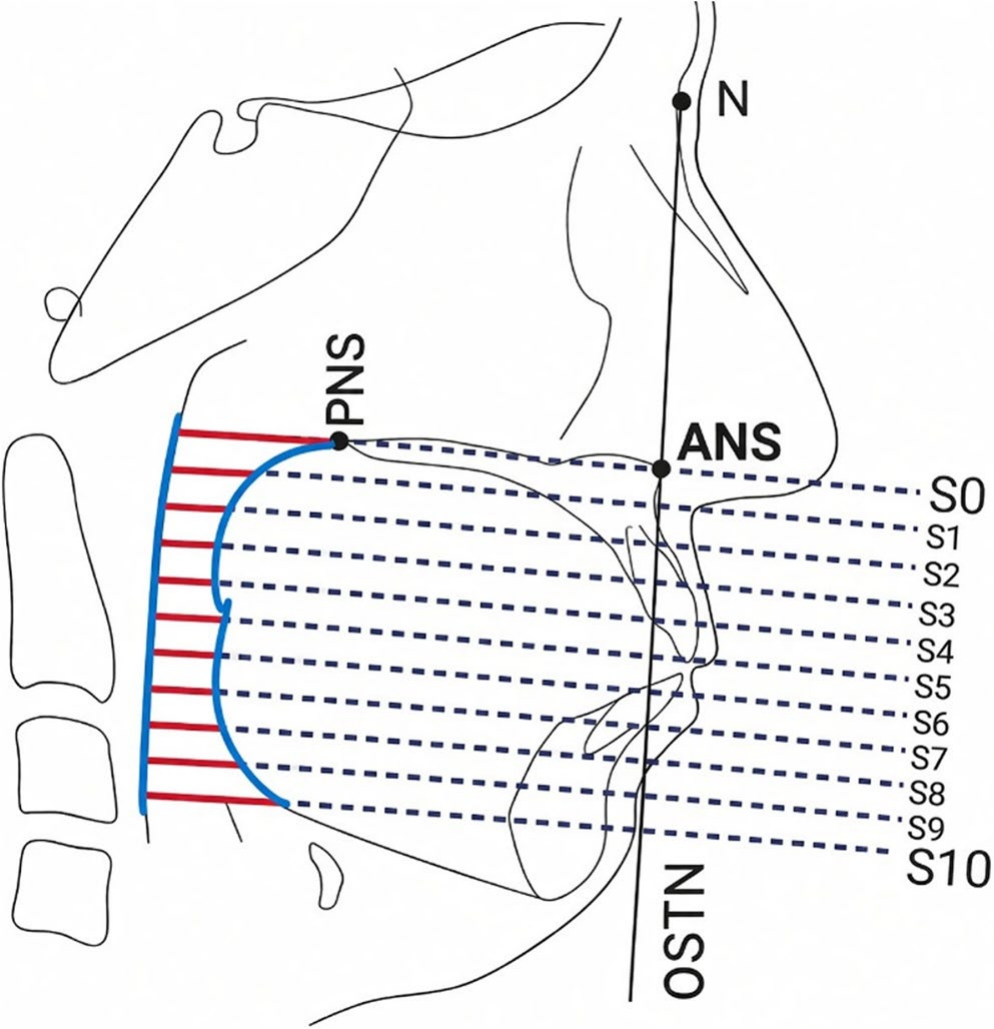
Cephalometric method used for pharyngeal airway analysis. The S0 plane (ANS–PNS) and the N⊥ plane (perpendicular to S0 through Nasion) are shown. The airway was divided into 11 parallel levels (S0–S10) from the velopharynx to the hypopharynx. Linear distances between the anterior and posterior pharyngeal walls were measured at each level. Adapted from Tsuiki et al. [25]

Upper airway dimensions were analyzed using the method of Tsuiki et al. [25], as illustrated in Fig 3. Two reference planes were defined: the S0 plane (ANS–PNS) and the N⊥ plane (perpendicular to S0 through Nasion). The airway was segmented into 11 parallel levels (S0–S10) at equal intervals from the upper border of the velopharynx to the base of the epiglottis. Linear distances between the anterior and posterior pharyngeal walls were measured at each level.

The superior boundary of the velopharynx was defined by the posterior extension of the S0 line, and the inferior boundary by a line passing through the tip of the soft palate, parallel to S0. The boundary between the oropharynx and hypopharynx was defined by a line passing through the tip of the epiglottis, also parallel to S0, while the inferior margin of the hypopharynx corresponded to the posterior extension of the S10 line. Accordingly, the airway was subdivided into the velopharynx (S0–S4), oropharynx (S4–S8), and hypopharynx (S8–S10).

PSG studies were performed in the Sleep Laboratory of the Department of Pulmonary Medicine, Istanbul University Faculty of Medicine, using the ALICE 5 system (Philips Respironics, USA). AASM (American Academy of Sleep Medicine) pediatric scoring was used, including EEG, EOG, EMG, oronasal airflow, oxygen saturation, respiratory effort, body position, and ECG [26]. Polysomnographic recordings were evaluated by pediatric pulmonology specialists, and management decisions were guided by interdisciplinary collaboration among orthodontists, pediatric pulmonologists, and otorhinolaryngologists.

Finally, the Pittsburgh Sleep Quality Index (PSQI) was used in the study. The PSQI is a 24-item questionnaire designed to assess changes in multiple dimensions of sleep over time. The instrument provides researchers and healthcare providers with a comprehensive assessment of an individual’s sleep patterns, and can be used to inform treatment decisions and interventions for sleep disorders [27].

### Statistical Analysis

The data were analysed using IBM SPSS v23. The normality of distribution was examined using the Shapiro-Wilk test. The primary outcomes of interest were changes in AHI, oxygen saturation parameters, and pharyngeal airway dimensions across time points. For the purpose of comparing dependent variables across time points, normally distributed quantitative data were analysed using Repeated Measures ANOVA. Post-hoc comparisons were conducted using the Bonferroni test. For the comparison of dependent variables between time points, non-normally distributed quantitative data were analysed using the Friedman test. Post-hoc comparisons were conducted using the Dunn test. Descriptive statistics for quantitative variables were presented as mean ± standard deviation and median (minimum-maximum). The significance level was set at *p* < 0.05. To evaluate measurement consistency, cephalometric radiographs were re-measured by the same examiner after a 2-week interval. Intra-observer reliability was assessed using intraclass correlation coefficients (ICC) and method error was calculated using Dahlberg’s formula.

## Results

The cephalometric variables at the follow-up (T3) visit compared with the pre- (T1) and post-treatment (T2) data are presented in Table 2. The initial treatment outcomes (T1–T2) for this cohort have been previously reported in the literature [23]. The differences between T2 and T3 were small and did not reach statistical significance except for Go-Me and 1-NA. Reliability analysis showed excellent repeatability, with ICC values of 0.90 or higher for all linear and angular measurements. Method error values ranged from 0.20 to 0.40 mm for linear variables and 0.25°–0.50° for angular variables.

**Table 2:** Comparison of cephalometric skeletal and dental parameters at T1 (pre-treatment), T2 (post-treatment), and T3 (7-year follow-up)

	T1	T2	T3	Test Statistic	*p*
SNA (°)	78.85 ± 3.89	78.23 ± 3.63	78.69 ± 3.64	1.778 ^x^	0.191
SNB (°)	71.54 ± 3.60 ^a^	74.54 ± 3.73 ^b^	74.00 ± 3.70 ^b^	26.469 ^x^	< 0.001
Go-Me (mm)	55 (52–71) ^a^	58 (55–74) ^b^	60 (56–76) ^c^	24.776 ^y^	< 0.001
ANS-PNS (mm)	48.46 ± 3.95 ^a^	51.38 ± 3.45 ^b^	51.77 ± 2.83 ^b^	32.318 ^x^	< 0.001
SN-GoMe (°)	40.38 ± 5.85	40.77 ± 6.46	40.54 ± 7.02	0.166 ^x^	0.750
1-SN (°)	108.54 ± 6.20 ^a^	100.85 ± 7.74 ^b^	104.46 ± 8.06 ^c^	21.567 ^x^	< 0.001
1-NA (mm)	6.08 ± 1.71 ^a^	4 ± 1.47 ^b^	4.62 ± 1.56 ^b^	13.445 ^x^	< 0.001
IMPA (°)	92.38 ± 6.97 ^a^	96.08 ± 8.61 ^b^	97.23 ± 7.35 ^b^	8.59 ^x^	0.002
1-NB (mm)	5.00 ± 1.83	5.77 ± 2.45	6.00 ± 2.38	1.906 ^x^	0.171
Overjet (mm)	8.5 (5.5–14) ^a^	2.5 (2–4) ^b^	3 (2–6) ^b^	21.745 ^y^	< 0.001
Overbite (mm)	4 (2.5-5) ^a^	2.50 (1.5–3.5) ^b^	2 (1–3) ^b^	20.043 ^y^	< 0.001

^x^ Repeated measures ANOVA; ^y^ Friedman test. Data are presented as mean ± standard deviation for normally distributed variables and median (range) for non-normally distributed variables. Values sharing the same superscript letter are not significantly different

Airway dimensions were assessed at 11 levels (S0–S10) and are presented in Table 3. At the velopharyngeal level, statistically significant increases were found at S0 (*p* = 0.002), with mean values rising from 13.46 mm (T1) to 14.85 mm (T3). At the same level, S3 also showed a significant change (*p* = 0.002), with values increasing at T2 and decreasing at T3. Within the oropharyngeal region (S6 and S8), significant increases were observed from T1 to T2 (*p* < 0.05), with no significant changes between T2 and T3. In the hypopharynx, S9 and S10 showed significant increases from T1 to T3 (*p* < 0.001), and no significant differences were observed between T2 and T3.

**Table 3:** Comparison of pharyngeal airway dimensions (S0–S10) at T1, T2, and T3

	T1	T2	T3	Test Statistic	*p*
S0	13.46 ± 3.31 ^a^	13.92 ± 3.93 ^a^	14.85 ± 3.31 ^b^	8.129 ^x^	0.002
S1	10.23 ± 3.72	10.69 ± 3.09	10.92 ± 3.01	2.066 ^x^	0.149
S2	8.69 ± 2.50	8.85 ± 2.38	8.69 ± 1.93	0.316 ^x^	0.732
S3	7.62 ± 2.26 ^ab^	7.92 ± 2.33 ^a^	7.38 ± 2.29 ^b^	8.222 ^x^	0.002
S4	7.38 ± 1.94	7.69 ± 1.97	7.77 ± 2.17	1.054 ^x^	0.339
S5	7 (6–13)	7 (6–13)	7 (5–12)	1.167 ^y^	0.558
S6	7 (6–10) ^a^	8 (7–11) ^b^	8 (7–11) ^b^	7.235 ^y^	0.018
S7	7.23 ± 1.09	7.38 ± 1.26	7.69 ± 1.75	1.240 ^x^	0.297
S8	6 (5–8) ^a^	7 (6–9) ^b^	7 (6–9) ^b^	8.473 ^y^	0.015
S9	7.15 ± 1.95 ^a^	8.08 ± 2.40 ^b^	8.77 ± 1.59 ^b^	15.857 ^x^	< 0.001
S10	10.15 ± 2.61 ^a^	11.46 ± 1.98 ^b^	12.15 ± 1.95 ^b^	17.385 ^x^	< 0.001

^x^ Repeated measures ANOVA; ^y^ Friedman test. Data are presented as mean ± standard deviation for normally distributed variables and median (range) for non-normally distributed variables. Values sharing the same superscript letter are not significantly different

Table 4 presents the AHI, lowest and mean oxygen saturation, sleep efficiency (%), arousal index, %REM sleep, and oxygen desaturation index (ODI) values at T1, T2, and T3. The percentage of REM sleep showed no significant change between T1 and T2, whereas the increase from T2 to T3 was statistically significant. Median AHI decreased from 2.3 events/hour at T1 to 0.5 events/hour at T2 (*p* < 0.05). At T3, AHI increased to 2.0 events/hour (*p* < 0.05).

**Table 4:** Comparison of polysomnographic parameters at T1, T2, and T3

	T1	T2	T3	Test Statistic	
Stage 1 (%)	0.5 (0.20–1.70)	0.3 (0.10–1.70)	0.5 (0.10–1.20)	4.542 ^y^	0.103
Stage 2 (%)	57.5 (25.50–74.70)	65.2 (22.90–70)	59.2 (41.70–78.90)	0.275 ^y^	0.872
Stage 3,4 (%)	30.6 (17.60–74)	28 (0-75.40)	25.3 (14.10–47.10)	1.846 ^y^	0.397
REM (%)	5.82 ± 5.04 ^a^	5.76 ± 3.83 ^a^	12.48 ± 5.50 ^b^	10.133 ^x^	0.002
AHI	2.3 (1-16.90) ^a^	0.5 (0.20–1.80) ^b^	2 (0.10–26) ^a^	12.980 ^y^	0.002
Mean saturation (%)	97.46 ± 0.78 ^a^	97.46 ± 0.97 ^a^	96.23 ± 1.36 ^b^	10.139 ^x^	0.002
Min. saturation (%)	92 (29–95)	92 (82–94)	89 (78–93)	3.040 ^y^	0.219
Sleep efficiency (%)	85.56 ± 8.29	88.15 ± 9.19	82.33 ± 11.96	1.808 ^x^	0.190
Arousal index	10.23 ± 7.69	13.49 ± 6.01	11.98 ± 7.57	0.533 ^x^	0.594
ODI	1.7 (0.60–5.90)	1.30 (0-3.10)	3 (0–18)	3.231 ^y^	0.199

*REM* Rapid eye movement, *AHI* Apnea–Hypopnea Index, *ODI* Oxygen Desaturation Index. ^x^ Repeated measures ANOVA; ^y^ Friedman test. Data are presented as mean ± standard deviation for normally distributed variables and median (range) for non-normally distributed variables. Values sharing the same superscript letter are not significantly different

Evaluations of PSQI scores are presented in Table 5. According to the scale, a total score of 5 or less indicates good sleep quality, while a score of 6 or more indicates poor sleep quality [28]. At T1, mean PSQI scores were within the range corresponding to good sleep quality. PSQI scores decreased at T2 and increased slightly at T3; however, these changes were not statistically significant, and mean values remained within the good sleep quality range [28].

**Table 5:** Comparison of Pittsburgh Sleep Quality Index (PSQI) scores and components at T1, T2, and T3

	T1	T2	T3	Test Statistic	*p*
Global score	5.23 ± 2.74 ^a^	3.15 ± 2.15 ^b^	3.85 ± 1.68 ^ab^	6.943 ^x^	0.005
Subjective sleep quality	1 (0–3)	0 (0–2)	1 (0–2)	5.688 ^y^	0.058
Sleep latency	1 (0–2)	1 (0–2)	1 (0–1)	1.040 ^y^	0.595
Sleep disturbances	1 (0–3)	1 (0–1)	1 (0–1)	3.263 ^y^	0.196
Daytime dysfunction	1 (0–3) ^a^	1 (0–3) ^b^	1 (0–3) ^ab^	7.032 ^y^	0.030

^x^ Repeated measures ANOVA; ^y^ Friedman test. Data are presented as mean ± standard deviation for normally distributed variables and median (range) for non-normally distributed variables. Values sharing the same superscript letter are not significantly different

Sleep duration, habitual sleep efficiency, and use of sleep medication components within the PSQI were excluded from comparisons, as all observations at each time point were identical and scored as zero

## Discussion

The aetiological factors of apnea in children include craniofacial anomalies, which play a significant role in influencing the dimensions of the airway [29, 30]. In the previous study, improvements were noted in mandibular position, overjet, and overbite, accompanied by decreased AHI scores, improved sleep quality, and reduced symptoms of SDB during both night and day [23].

The mean AHI value of 2.0 at T3 indicates persistence of SDB, despite participants having transitioned into late adolescence or early adulthood at follow-up. The observed increase over time suggests that long-term functional control was not fully maintained. According to current pediatric sleep medicine guidelines, including those from the American Academy of Sleep Medicine, even mild elevations in AHI may be clinically relevant in children and adolescents, underscoring the importance of cautious interpretation of longitudinal changes [31].

In addition to changes in AHI, several polysomnographic parameters demonstrated unfavorable trends at long-term follow-up. Sleep efficiency showed a tendency to decrease, arousal-related indices increased, and a significant reduction in mean oxygen saturation values was observed at T3 (*p* = 0.002). Although minimum oxygen saturation values did not decline to clinically critical levels, the presence of these unfavorable changes underscores the heterogeneous and non-linear evolution of SDB during adolescence. These findings indicate that long-term outcomes following pediatric OSA treatment may involve concurrent improvements and deteriorations across different sleep domains, rather than a uniform trajectory of sustained improvement.

A statistically significant increase in REM sleep percentage was observed at T3. Although this increase occurred concurrently with a rise in AHI, REM sleep percentages at T1 and T2 were relatively low compared to expected values for this age group. Previous studies have reported that REM sleep may be influenced by recording conditions and developmental factors, and that REM proportions tend to increase and stabilize during adolescence [32, 33]. However, the underlying mechanisms of the observed increase in REM sleep in the present study cannot be determined, and this finding should therefore be interpreted cautiously, without attributing it to a specific cause.

The PSQI, a self-report questionnaire designed to assess subjective sleep quality [27], showed improvement between T1 and T2, in parallel with objective PSG findings. However, between T2 and T3, despite a statistically significant increase in AHI, PSQI scores remained unchanged, suggesting a complex and potentially discordant relationship between objective and perceived sleep outcomes. Previous studies have similarly reported that a significant correlation between AHI and PSQI is not consistently observed, particularly in patients with mild to moderate SDB, who may still perceive their sleep quality as satisfactory [34]. These findings should be interpreted within the exploratory context of the present study, with greater emphasis on longitudinal patterns rather than isolated statistically significant differences.

The cephalometric analysis indicated relative stability in skeletal parameters between T2 and T3, with no statistically significant differences except for Go-Me and 1-NA. The absence of significant changes in most skeletal variables during the follow-up period suggests a degree of maintenance of the post-treatment skeletal configuration over time, which is consistent with findings reported by Han et al. [13] and Godt et al. [12], who documented long-term skeletal stability following growth-modifying treatments in Class II patients. The increase in Go-Me distance between T2 and T3 is consistent with expected mandibular growth during adolescence. However, given the inherent limitations of two-dimensional cephalometric analysis, no direct inference can be made regarding associated soft tissue or muscular adaptations.

The pharyngeal airway analysis is consistent with the possibility of sustained airway dimensional changes following treatment. Significant and sustained increases in oropharyngeal and hypopharyngeal dimensions (particularly at S6, S8, S9, and S10) were observed, suggesting that airway dimensions remained increased compared to baseline over the 7-year period. Interestingly, the velopharyngeal region (S0) demonstrated a gradual increase, whereas the hypopharyngeal level (S3) showed a decrease from T2 to T3, returning to near-baseline levels. This regional variation may reflect the differential response of soft tissues and neuromuscular control mechanisms in distinct anatomical zones of the airway. These findings are consistent with those reported by Temani et al. [35] and Yue et al. [36], who demonstrated short-term increases in oropharyngeal and hypopharyngeal airway dimensions following mandibular advancement, though both studies lacked long-term follow-up data.

In a longitudinal cephalometric study in which subjects presenting with Class I skeletal patterns and only minor orthodontic intervention were selected as a control group for Class II patients treated with activator-headgear therapy, sagittal pharyngeal airway dimensions demonstrated gradual increases during growth; however, the magnitude of these changes was generally limited and not uniformly distributed across all airway levels [14]. These findings suggest that physiologic growth alone results in modest, region-specific airway enlargement rather than marked or generalized expansion, lending contextual support to the interpretation that the more pronounced airway changes observed in the present cohort may reflect, at least in part, the effect of mandibular advancement therapy.

Systematic reviews and meta-analyses published in recent years have synthesized the available evidence on the effectiveness of mandibular advancement appliances in children. A meta-analysis of randomized and non-randomized trials reported a significant reduction in AHI compared to control in children treated with mandibular advancement appliances [16], while an umbrella review confirmed that AHI improvement is a consistent finding across existing systematic reviews, though randomized controlled evidence remains limited [17]. A further systematic review evaluating combined mandibular advancement and maxillary expansion similarly reported reductions in AHI and improvements in oxygenation [18]. These findings are supported by a broader body of individual studies documenting beneficial effects of mandibular advancement devices, including the monoblock appliance, on airway dimensions and sleep-related parameters [10, 23, 37–42]. Studies using cephalometry and cone beam computed tomography (CBCT) have demonstrated structural airway enlargement following mandibular advancement, although findings vary across appliance types and populations [12–14, 41, 43]. Notably, recent prospective work combining cephalometric, CBCT, and polysomnographic assessment concurrently has provided simultaneous structural and functional evidence [42]. Studies specifically assessing long-term effects in pediatric populations remain scarce [12, 15, 44]. In this context, the present study extends the available evidence by combining cephalometric and PSG assessments across a 7-year follow-up in a cohort of children with mandibular retrognathia — a duration and dual-domain design that is uncommon in the existing literature.

In contrast, studies incorporating polysomnography alongside structural assessment to evaluate functional outcomes of mandibular advancement in children are more limited in number [20–22, 42–45]. Among those combining both domains, Mastud et al. [42] assessed treatment outcomes using cephalometric, CBCT, and polysomnographic measurements concurrently in a prospective cohort of pediatric OSA patients, providing simultaneous structural and functional evidence. Similarly, studies by Cortes-Mejia et al. [20], Kim et al. [21], and Marcussen et al. [22] combined structural and polysomnographic or airway outcomes, contributing relevant evidence on the relationship between anatomical changes and functional improvement, though they differ from the present study in patient age, appliance type, and follow-up duration. For example, while Pirelli et al. [45] demonstrated sustained polysomnographic improvements over a 12-year follow-up, that study focused on children with isolated maxillary constriction rather than mandibular retrognathia. In contrast, the present cohort was defined by mandibular retrognathia and polysomnographically confirmed OSA, and the 7-year combined assessment revealed that cephalometric airway dimensions remained increased at T3 while AHI showed a statistically significant partial recurrence — a divergence between structural and functional outcomes that is not readily captured by studies examining only one domain.

Taken together, prior literature on this topic falls into two related but distinct strands. The first focuses on craniofacial or airway morphology, where mandibular advancement appears to produce structural enlargement during the treatment period, although findings are heterogeneous and often short-term [12, 14, 16, 17, 41–43]. The second focuses on functional outcomes measured by polysomnography, where reductions in AHI and improvements in oxygenation have been reported in selected cohorts, but durable normalization across the growth period remains inconsistent [10, 18, 39, 45, 46]. The present cohort contributes to this evidence base by combining both domains over a 7-year period: while cephalometric changes were relatively stable, SDB partially recurred during adolescence. Importantly, the observed structural enlargement on cephalometric analysis did not correspond to sustained functional control of SDB — a dissociation consistent with current concepts in sleep medicine recognizing that upper airway patency is determined not only by anatomical dimensions but also by neuromuscular control, ventilatory stability, and arousal threshold [47]. Given that childhood OSA is increasingly understood as a dynamic disorder influenced by structural, neuromuscular, and behavioral factors [19, 48, 49], this combined evaluation underscores that structural improvements, while maintained, may be insufficient on their own to ensure long-term functional stability.

Several limitations of the present study must be acknowledged. Given the longitudinal nature of the study and the absence of an untreated control group, the observed craniofacial and airway changes should be interpreted within the context of normal growth and maturation. Participants transitioned from late childhood into adolescence and early adulthood during the follow-up period, a developmental stage characterized by significant skeletal, neuromuscular, and airway-related changes. It is therefore not possible to isolate treatment-related effects from physiological growth processes, and the findings likely reflect the combined influence of both factors. Although an ideal comparison would include untreated Class II individuals, withholding indicated orthopedic intervention in growing patients with confirmed OSA raises ethical concerns, rendering direct comparison with an untreated cohort infeasible.

The assessment of upper airway dimensions was based on lateral cephalometric radiographs, which provide a two-dimensional representation of a complex three-dimensional structure and do not capture volumetric or dynamic airway changes. Consistent with recent evidence, specific cephalometric parameters — including mandibular retrognathia, hyoid bone position, and cranial base angle — show statistically significant associations with pediatric OSA; however, lateral cephalometry cannot reliably serve as a standalone diagnostic tool for sleep-disordered breathing in children [50]. Furthermore, two-dimensional projections do not capture the three-dimensional volume or dynamic collapsibility of the pharyngeal airway, which limits the interpretation of the absolute dimension values reported in the present study. Future studies incorporating CBCT or MRI-based airway analysis may allow more accurate volumetric and functional assessment. Nevertheless, the use of a standardized cephalometric protocol and a consistent imaging system across all time points supports the internal validity of within-subject longitudinal comparisons, allowing for reliable evaluation of relative changes over time.

Several aspects of phenotypic characterization were not systematically recorded. Although all patients underwent otorhinolaryngological evaluation prior to enrollment — with those exhibiting clinically significant adenotonsillar hypertrophy referred for surgical management and not enrolled — adenotonsillar grading, nasal patency, positional dependency, and other upper airway contributors were not incorporated into the study database for quantitative analysis. Therefore, the relative contribution of mandibular retrognathia versus other upper airway factors to the observed outcomes cannot be determined, and caution is warranted in attributing the findings exclusively to mandibular advancement therapy. Future studies should incorporate systematic phenotypic characterization at baseline to allow more granular attribution of outcomes.

The exclusion of participants who developed obesity at follow-up represents a further source of attrition bias. Although these individuals did not differ from the included participants at baseline in terms of age, craniofacial characteristics, or initial AHI values, their exclusion may have resulted in a more favorable representation of long-term outcomes, as obesity is a well-established risk factor for worsening OSA in adolescence. The final cohort may therefore represent a selected subgroup with a more favorable developmental trajectory, and the results should be interpreted accordingly.

Despite these limitations, the 7-year follow-up period represents a notable strength of the study, as extended longitudinal data combining polysomnographic and cephalometric assessments in pediatric OSA remain limited in the literature. The use of standardized PSG, validated questionnaires, and a consistent cephalometric protocol across all time points further supports the internal reliability of the findings. Nevertheless, larger prospective and multicenter studies are required to confirm these observations and improve generalizability.

From a clinical perspective, the results are consistent with the possibility that monoblock mandibular advancement therapy may play a role in skeletal and airway adaptation in selected children with mandibular retrognathia and OSA. However, variability in airway response and the partial increase in AHI observed during adolescence indicate that outcomes are not uniform and that long-term follow-up remains essential. These findings should be considered within a multidisciplinary management framework, given that diagnosis, treatment planning, and follow-up of pediatric OSA require coordinated collaboration among orthodontists, pediatric pulmonologists, and otorhinolaryngologists. Although cephalometric parameters are not routinely applied in otolaryngologic practice, they provide structural insight into craniofacial morphology and its relationship with upper airway configuration. When interpreted alongside polysomnographic findings, such measurements contribute to a more comprehensive understanding of the structural and functional adaptations associated with mandibular advancement therapy.

In conclusion, this long-term follow-up study provides a descriptive evaluation of skeletal, airway, and sleep-related changes in children with mandibular retrognathia and OSA treated with a monoblock appliance. Mandibular positioning and pharyngeal airway dimensions remained relatively stable over the 7-year observation period, while a mild but statistically significant increase in AHI was observed during adolescence, indicating a degree of functional recurrence despite the maintenance of structural changes. These findings reflect the dynamic and multifactorial nature of pediatric OSA and underscore the need for sustained, individualized monitoring over time. The study design does not permit strong causal inference regarding treatment effects, and the observed changes likely reflect a combination of intervention-related factors, normal growth and maturation, and other time-dependent developmental processes. Further multicenter longitudinal studies incorporating systematic phenotypic characterization and validated functional outcome measures are warranted to clarify these relationships.

## Data Availability

The datasets generated and analyzed during the current study are available from the corresponding author upon reasonable request.
